# Blinatumomab Maintenance Therapy Following Bone Marrow Transplantation for Early Relapsed Pediatric B-cell Precursor Acute Lymphoblastic Leukemia and Analysis of Lymphocyte Subset Changes

**DOI:** 10.7759/cureus.62263

**Published:** 2024-06-12

**Authors:** Takanari Abematsu, Takuro Nishikawa, Hiroshi Kasabata, Shunsuke Nakagawa, Yasuhiro Okamoto

**Affiliations:** 1 Department of Pediatrics, Graduate School of Medical and Dental Sciences, Kagoshima University, Kagoshima, JPN; 2 Department of Clinical Laboratory Medicine, Kagoshima University Hospital, Kagoshima, JPN

**Keywords:** hematopoietic stem cell transplantation, naïve t cell, maintenance, blinatumomab, acute lymphoblastic leukemia

## Abstract

Blinatumomab, a CD19/CD3 bispecific T-cell engager, is recognized as an effective immunotherapy for relapsed B-cell precursor acute lymphoblastic leukemia (BCP-ALL). However, the efficacy and safety of blinatumomab in post-hematopoietic stem cell transplantation (HSCT) maintenance therapy has not been established. A 5-year-old male patient with BCP-ALL suffered a relapse in his bone marrow during maintenance therapy. After re-induction therapy with UK-R3 regimen, 2.3% of the blasts remained. Then the blinatumomab was administered, and he achieved minimal residual disease (MRD)-negative complete remission (CR). After two cycles of blinatumomab, he underwent allogeneic bone marrow transplantation (BMT) from his human leukocyte antigen (HLA)-matched sibling, following conditioning with total body irradiation, etoposide, and cyclophosphamide. Two cycles of blinatumomab maintenance therapy were initiated to prevent relapse. There was no exacerbation of graft-versus-host disease (GVHD) or other severe adverse events. CR was maintained for >22 months after BMT. A t-distributed symmetric neighbor embedding (tSNE) analysis revealed that blinatumomab altered the CD8+ population, as with pre-HSCT use, and markedly reduced the CD8+19dim+/CD8+CD19- ratio (i.e., naïve lymphocyte predominance). Blinatumomab maintenance therapy after HSCT may be considered a safe treatment.

## Introduction

Acute lymphoblastic leukemia (ALL) is the most common childhood malignancy. Currently, 85% of childhood ALL patients are cured, and overall survival (OS) rates are up to 90% with standard chemotherapy according to risk stratification [1,2]. The most common cause of treatment failure in childhood ALL is relapse, which occurs in approximately 15-20% of patients and cure rates are much lower after relapse [3]. Survival of relapsed patients can be predicted by the site of relapse, time to relapse, and the immunophenotype of relapsed ALL. Early bone marrow relapse is classified as high risk in the Berlin-Frankfurt-Münster (BFM) stratification and is a significant predictor of poor event-free survival (EFS) [3,4]. Long-term prognosis in pediatric patients with high hyperdiploid B-cell precursor (BCP)-ALL and early bone marrow relapse is poor even if a second remission is achieved, and it has a second remission rate of 86%, 5-year OS rate of 29% (95% confidence interval (CI): 8-52%), and 5-year EFS rate of 21% (95% CI: 5-45%) [5]. In addition, the presence of minimal residual disease (MRD) at the end of induction and consolidation therapy predicts a poor prognosis, even if the patient receives an allogeneic hematopoietic stem cell transplant (HSCT) because the patient is more likely to relapse than those with MRD-negative disease.

Blinatumomab is a bispecific T cell-engaging antibody construct that links CD3+ T cells to CD19+ leukemia cells and induces a cytotoxic immune response. Blinatumomab has been reported to be advantageous in terms of survival, MRD-negative rate, and minimal therapy-related toxicity such as cytopenia, severe infections, and sepsis compared with chemotherapy alone in pediatric patients with relapsed and refractory (R/R) BCP-ALL [6-8]. However, the efficacy and safety of blinatumomab in post-HSCT maintenance therapy have not been established.

Informed consent was obtained from the patient and his parents to publish this case report. The case report was approved by the Ethics Committee on Clinical Research, Sakuragaoka Campus, Kagoshima University (approval number: 230260). This article was previously presented as a meeting abstract at The 65th Annual Meeting of the Japanese Society of Pediatric Hematology/Oncology on September 29, 2023.

## Case presentation

A 5-year-old boy exhibited high hyperdiploid BCP-ALL relapse in the bone marrow during maintenance therapy; ALL was first diagnosed at 2 years and 10 months old. Flow cytometry of the bone marrow aspirate at relapse showed positive results for HLA-DR, CD10, CD19, CD22, CD34, and CD38 expression levels, similar to those at first presentation. According to BFM stratification, early bone marrow relapse was diagnosed [3]. The patient underwent remission induction therapy with the ALL R3 regimen [9]. During the second week of remission induction therapy, the patient developed pancreatitis (Common Terminology Criteria for Adverse Events (CTCAE) grade 3) and sepsis (CTCAE grade 3), and treatment was paused for 4 weeks and then resumed. At the end of induction therapy, bone marrow examination showed 2.3% of blasts remaining. One cycle of blinatumomab was administered, and polymerase chain reaction-based MRD-negative complete remission (CR) was achieved. CTCAE grade 1 cytokine release syndrome (CRS) occurred immediately after blinatumomab administration but was quickly resolved. After two cycles of blinatumomab, the patient received allogeneic bone marrow transplantation (BMT; nucleated cell counts 6.2×108 /kg) from his human leukocyte antigen-matched sibling, followed by conditioning with total body irradiation (2 Gy × 6), etoposide (60 mg/kg), and cyclophosphamide (60 mg/kg × 2). Cyclosporine A was administered as prophylaxis for graft-versus-host disease (GVHD). Neutrophil engraftment was achieved on day 11. Stage 3 skin and no gastrointestinal or liver total Grade II acute GVHD were exhibited on day 14; however, these improved with topical steroid application. To prevent relapse, two cycles of blinatumomab maintenance therapy were initiated on days 40 and 81. Similar to blinatumomab administration prior to BMT, blinatumomab induced a swift decline in T cell counts upon initiation of infusion, followed by recovery. Adverse events included CRS (CTCAE grade 1) and neutropenia (CTCAE grade 3). However, these adverse events did not affect the continuation of blinatumomab therapy. No exacerbations of GVHD or other severe adverse events were observed. CR was maintained after BMT for >22 months.

**Figure 1:**
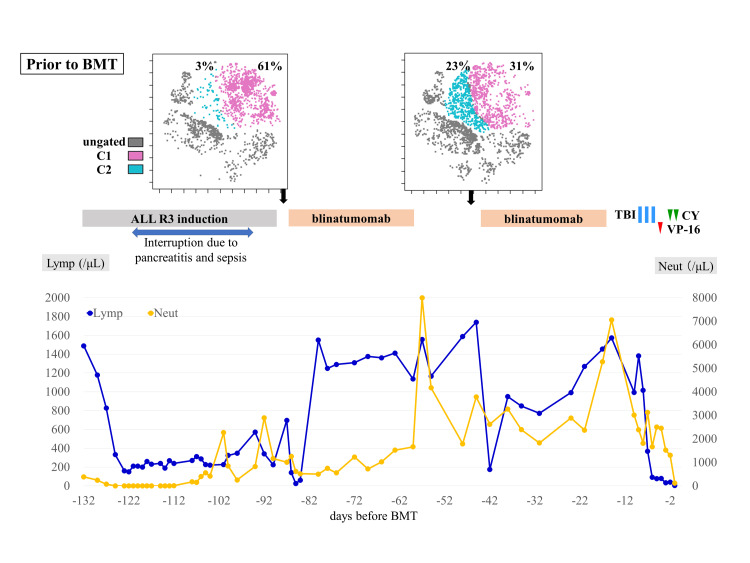
Treatment course prior to BMT and changes in CD8-positive cell populations determined by tSNE-CUDA analysis. BMT: bone marrow transplantation; ALL: acute lymphoblastic leukemia; TBI: total body irradiation; CY: cyclophosphamide; VP-16: etoposide; Lymp: lymphocyte count; Neut: neutrophil count; tSNE-CUDA: t-distributed stochastic neighbor embedding. tSNE-CUDA analysis was performed at each time point and the percentage of C2 cells increased following the first course of blinatumomab. The image is drawn by the authors of this article.

**Figure 2:**
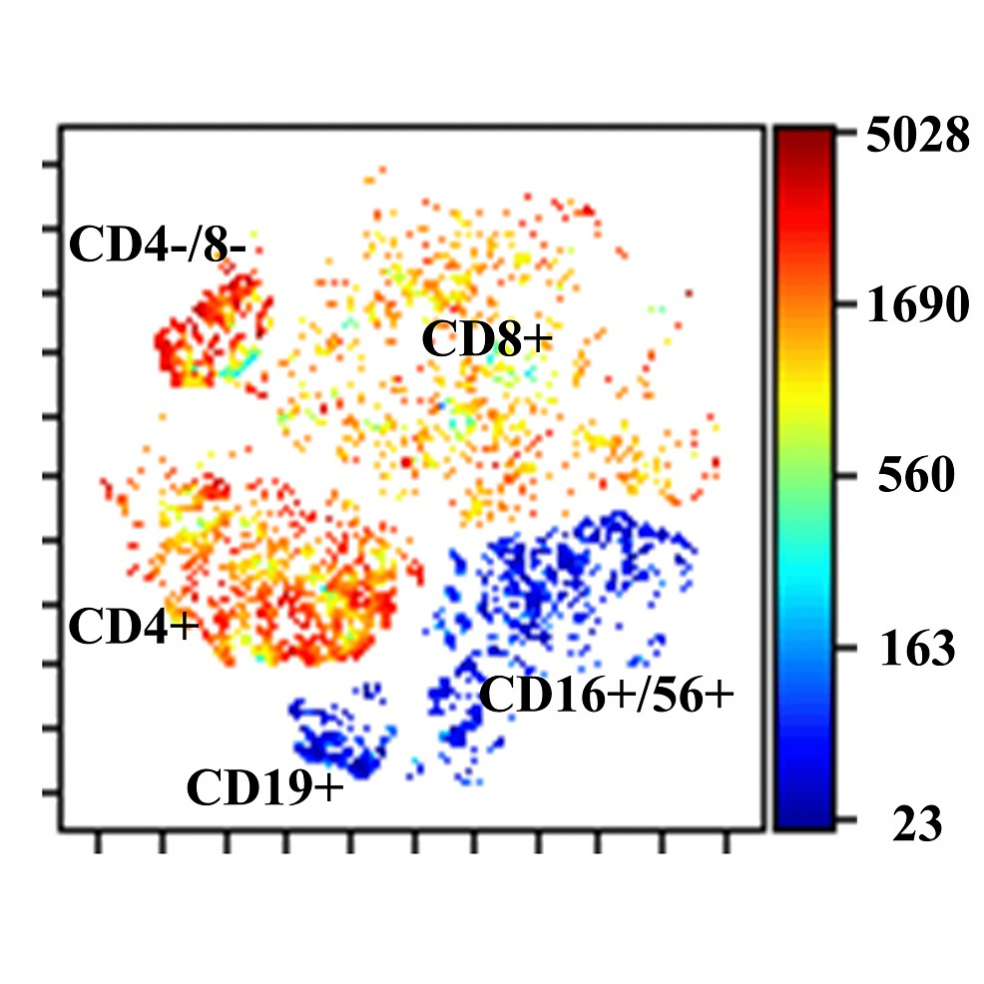
Distribution of lymphocyte subgroups determined by tSNE-CUDA analysis of the patient. This study focused on the CD8-positive cell population. The image is drawn by the authors of this article. tSNE-CUDA: t-distributed stochastic neighbor embedding.

Peripheral blood mononuclear cells were analyzed chronologically using flow cytometry with six colors (CD45, CD3, CD16/CD56, CD4, CD19, and CD8). Flow cytometry standard files were subjected to graphics processing units (GPU)-accelerated t-distributed stochastic neighbor embedding (tSNE-CUDA) analysis using the Cytobank program. tSNE-CUDA analysis revealed that blinatumomab altered the CD8-positive cell population in peripheral blood (Figures 1-2).

**Figure 3:**
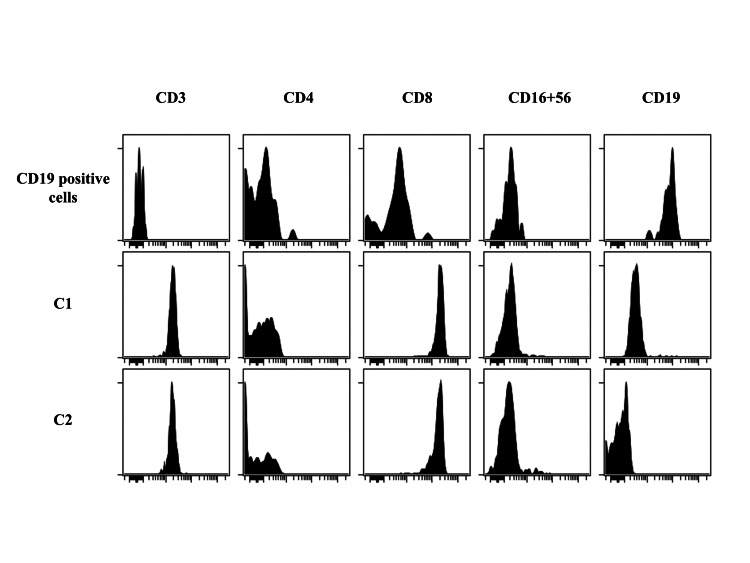
Analysis of surface marker expression of the patient. Differences in CD19 expression between C1 and C2 stages. The image is drawn by the authors of this article.

**Figure 4:**
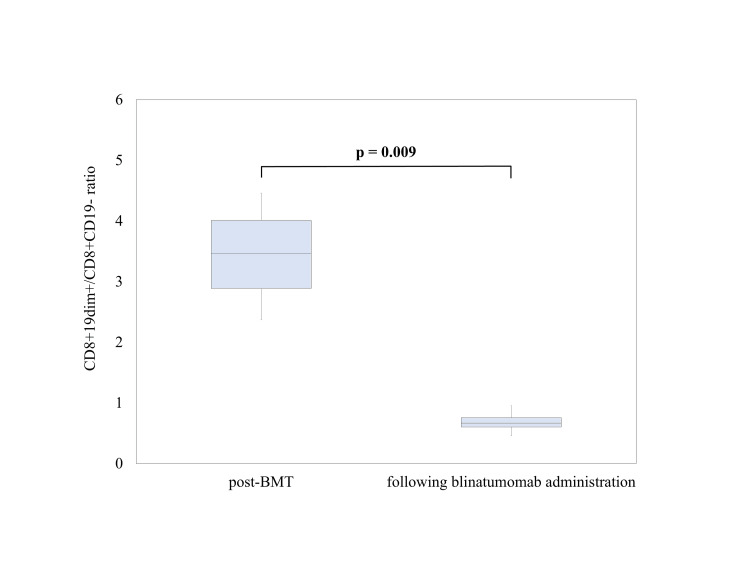
Comparison of CD8+19dim+/CD8+CD19 ratios after transplantation and following administration of blinatumomab. The boxplot shows the CD8+19dim+/CD8+CD19- ratio post-BMT after blinatumomab administration. Post-BMT included four time points from day 18 to 39 post-transplant, and following blinatumomab administration included six time points from day 8 to 88 after initiation of post-transplant blinatumomab. Welch’s t-test showed that the CD8+19dim+/CD8+CD19- ratio was significantly lower after blinatumomab administration than post-BMT (p = 0.009). The image is drawn by the authors of this article.

**Figure 5:**
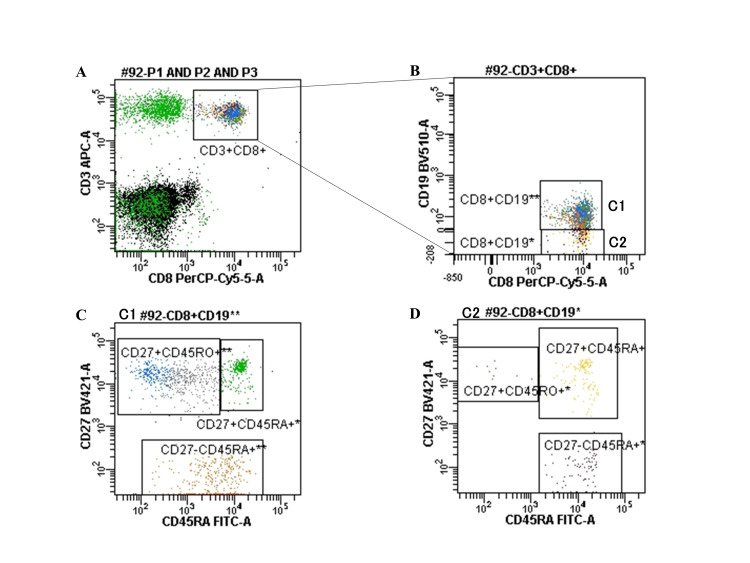
Flow cytometric analysis of healthy sample. (A) CD3 and CD8-positive cells were isolated. (B) CD8-positive cells were classified into C1 and C2 based on the intensity of CD19 expression. (C) C1 shows a balance between memory and naïve T cells. (D) C2 contains some memory T cells and is dominated by naïve T cells. The image is drawn by the authors of this article.

**Figure 6:**
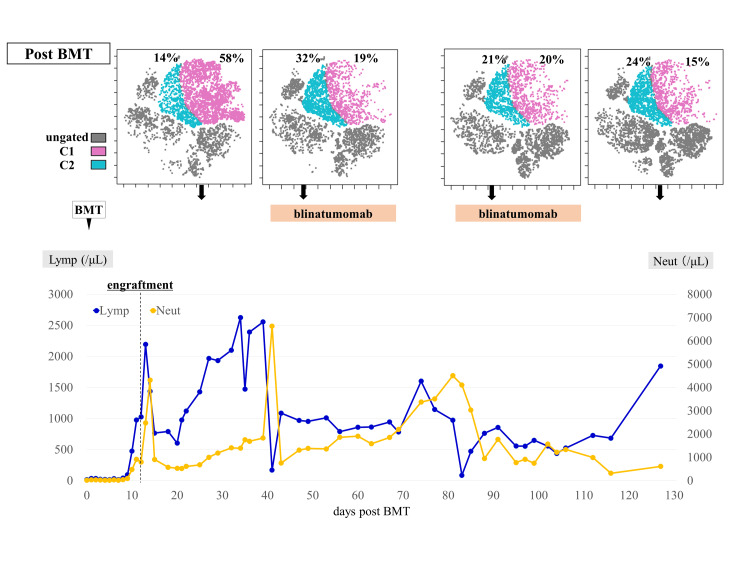
Treatment course post-BMT and changes in CD8-positive cell populations determined by tSNE-CUDA analysis. BMT: bone marrow transplantation; Lymp: lymphocyte count; Neut: neutrophil count; tSNE-CUDA: t-distributed stochastic neighbor embedding. tSNE-CUDA analysis was performed at each time point. After BMT, the percentage of C1 increased, and following post-transplant blinatumomab administration, the percentage of C2 increased compared to pre-BMT levels. The image is drawn by the authors of this article.

To identify the changes in cell populations, the expression of each surface marker was analyzed using patient samples of CD8-positive cells, with C1 and C2 as the right- and left-located cell populations, respectively. C1 was CD19dim+ and C2 was CD19-negative (Figure 3). Blinatumomab markedly reduced the CD8+CD19dim+/CD8+CD19-ratio (Figure 4). Analysis of healthy control samples (n = 3) showed that the population of CD8+CD19dim+ cells contained an even distribution of memory and naïve T cells, whereas the population of CD8+CD19-cells contained few memory T cells and was dominated by naïve T cells (Figure 5). In other words, the percentage of naïve T cells increased after treatment with blinatumomab, and the increase showed the same trend both pre- and post-BMT (Figure 6). FCM analysis showed that the percentage of T cells was CD8+CD45RA+ 38% and CD8+CD45RO+ 11% at 6 months post-transplant, CD8+CD45RA+ 42% and CD8+CD45RO+ 11% at 12 months post-transplant, and CD8+CD45RA+ 44% and CD 8+CD45RO+ 5% at 22 months post-transplant. To date, the observed trend of naïve T cells predominating above memory T cells has persisted.

## Discussion

Our patient responded very well to blinatumomab therapy, and then naïve T cells proliferated after blinatumomab treatment. This significant proliferation of naïve T cells suggests that blinatumomab not only targets leukemic cells but also affects the patient’s immune system, potentially contributing to its efficacy in maintaining remission [10]. The mechanisms behind blinatumomab-induced immune reconstitution involve both direct and indirect effects on T cells. Blinatumomab directly activates T cells by bringing them into close proximity with their target cells, causing T cells to have a killing effect on leukemia cells. Following this activation, a new cohort of T cells expands [11]. The reconstitution of a healthier immune profile, characterized by a higher proportion of naïve T cells, may enhance the patient’s ability to mount an effective immune response against residual leukemic cells [10]. The shift from memory to naïve T cells indicates a resetting of the immune system, which could be advantageous in preventing relapse by reducing the likelihood of residual malignant cells escaping immune surveillance. This is particularly important in the post-transplant setting, where the patient’s immune system is recovering from the intense conditioning regimen and the transplant itself. Restoration of naïve T cells earlier in the post-transplant period, when lymphocytes are depleted, is considered advantageous in terms of the graft-versus-leukemia effect and immunity to infection [10]. Changes in lymphocyte subsets may prove useful in predicting blinatumomab responses [11,12]. For instance, the observed shift in the CD8+ T cell population from memory T cell dominance to naïve T cell dominance could serve as a biomarker for evaluating the efficacy of blinatumomab therapy. It is reported that the median number of naïve CD8+ T cells in the responder group increased by 250% from the pre-treatment level at the end of the first cycle of blinatumomab treatment [12]. If future studies confirm these findings, monitoring lymphocyte subset changes could become a standard part of assessing patient response to blinatumomab, enabling more personalized and effective treatment strategies. A deeper understanding of the impact of blinatumomab on lymphocyte dynamics post-transplant could provide valuable insights into the optimization of maintenance therapy regimens and the improvement of long-term outcomes for pediatric patients with high-risk BCP-ALL. Further studies are necessary to elucidate the relationship between blinatumomab efficacy and changes in lymphocyte subsets, and to confirm the safety and effectiveness of this approach in larger pediatric cohorts.

New therapeutic strategies are needed to reduce the risk of relapse in patients with high-risk BCP-ALL. The use of blinatumomab as maintenance therapy post-HSCT is a promising approach that addresses the critical need for effective relapse prevention. In a single-center phase II study of relapsed refractory B-ALL including adolescents and young adults, blinatumomab maintenance therapy after HSCT was well tolerated, with a 1-year OS rate of 85% (95% CI; 61-95%) and 1-year progression-free survival rate of 71% (95% CI; 47-86%) [13], which is the only report of post-transplant blinatumomab therapy, and there are no reports of its use in children. In our case, post-transplant blinatumomab maintenance therapy was administered with the intention of improving the prognosis and was safe, with no serious adverse events, including GVHD exacerbation. This finding is of critical importance, as it opens the possibility of incorporating blinatumomab into post-transplant maintenance protocols for pediatric patients, who may benefit from its targeted action and low toxicity profile. Phases I and II trials of pediatric R/R B-ALL are ongoing (JPLSG SCT-ALL-BLIN21) in Japan [14]. These trials will provide more comprehensive data on the safety and efficacy of blinatumomab in the pediatric population. The incorporation of blinatumomab into post-transplant maintenance therapy regimens could represent a significant advancement in the treatment of high-risk pediatric BCP-ALL, offering hope for improved survival and quality of life for these patients.

## Conclusions

In our case, blinatumomab maintenance therapy following allogeneic HSCT in a pediatric patient was safe. The percentage of naïve T cells apparently increased after blinatumomab administration, and the increase showed the same trend pre- and post-BMT. The observed increase in naïve T cells post-treatment suggests a beneficial reconstitution of the immune system, which may contribute to the prevention of relapse. Further studies are needed to clarify the relationship between the efficacy of blinatumomab and changes in lymphocyte subsets before and after blinatumomab administration, and to confirm the safety and effectiveness of this approach in larger pediatric cohorts.
